# On the Relation of Dentist and Patient

**Published:** 1874-09

**Authors:** F. W. Sage

**Affiliations:** Sandusky, O.


### ON THE RELATION OF DENTIST AND PATIENT.

BY F. W. SAGE, SANDUSKY, O.

Of all the professions, we affirm without fear of a demurrer, there is none which take precedence of the dental profession as regards the importance and necessity for the maintenance of a dignified, affable and courteous address on the part of the practitioner, as an element of success in an effort to gain the confidence and respect of his patrons. Whatever may be his professional acquirements, however proficient he may have become in a knowledge of all the minutiae of dental science and in the ability to adapt and apply that knowledge in practice; whatever may be his manipulative ability; all these excellencies in combination can never make any man entirely independent of the impression, favorable or unfavorable which his personal presence effects in the minds of his patients.

In no other profession is the practitioner more constantly brought into direct personal contact with his patrons, and in no other profession does he sustain a relation more intimately confidential. In view, then, of this fact, we are not suprised to find, in many instances, that there are considerations arising in the mind of the individual who seeks the services of a dentist, which are entirely foreign to the question of his ability to perform the operations required, and which look more particularly to the individual characteristics and native disposition of the man. No one more fully realises or is more keenly appreciative of this fact, this phase of human nature, than is the charlatan. It is a notorious fact and one which appears to be of almost universal application, that the successful quack (success we mean in the sense of ability to secure patronage) owes his success, in a great measure, to a studiously cultivated suavity, or some other impalpable and indescribable personal quality or combination of qualities. So important does he consider it to be that nothing shall be done which shall in any degree detract from the favorable impression which he seeks first of all to stamp on the mind of the patient, that he will without scruple, jeopardize the integrity of an operation in cases where its failure at some remote future period will not in all probability be attributed to its true causes; rather than inflict pain, or it may be subject the patient to the tedium of a prolonged sitting. Thus it comes to pass that he not infrequently gains a wide-reaching reputation for careful and gentle manipulation, and the number of his patients increases in a ratio which fully compensates for the loss of patronage of those who finally awaken to a realising sense of his utter incompetency.

No very acute power of discernment is required to enable us to determine what qualities in a man recommend him to the public. Granted that he is *au fait* in the principles and practical details of his profession, it is obviously indispensable to his successful establishment in practice or to the retention of patronage when established, that he should cultivate many qualities which are readily suggested, as the following, to wit: Personal cleanliness, a regard for the comfort and convenience of his patient, gentleness of manipulation and the avoidance of the infliction of little annoyances not to say actual pain, so far as compatible with the requirements of the operation to be performed. Not to enumerate further, the idea which it is desirable should be impressed upon all may be briefly expressed in two words, cultivate tact. The fact that as is usually the case a large majority of our patients are ladies, lends an additional importance to the subject. It is not always the case that the masculine mind is capable of an adequate appreciation of the delicacy of feeling, the shrinking timidity and modesty, the squeamishness, if you please, (which whether it be unreasonable or not, it is not our present purpose to consider) prevails largely among women. Though these characteristics are not always conspicuously present their existence may be resonably inferred.

It behoves the dentist then to be mindful of the peculiarities of the sex and to be tolerant of the failings and idiosyncrasies of *any* and *all* for whom he may operate, carefully repressing all hasty impulses, preserving a quiet gentlemanly deportment, and endeavoring by a gentle yet firm manner to gain the *confidence* of the patient which being achieved, he may then and, not until then, expect to secure his consent to the performance of any operation which may be necessary. It is well to remember that the majority of persons have an exaggerated notion of the terrors of dental operations. In vain shall we expect to disabuse them of this impression by ridicule or disavowal. The attempt will be regarded as evidence of a lack of sympathy and appreciation. Rather let our efforts be directed to assure them of our fullest sympathy and appreciation and of our purpose to avoid all unnecessary infliction of pain.

It is not perhaps an unfeeling remark which some one has made that the general surgeon evinces the most admirable tact who *manifests* the largest degree of sympathy with the patient and *feels* the least. The opinion is probably based on the assumption that too much genuine sympathy incapacitates for the proper performance of the operation. If the principle applies to the general surgeon may it not as fitly be applied to the dentist?

Much judgment and tact is required on the part of the dentist in many other matters arising from his relation to the patient. It will frequently be necessary for him to make a dignified firm assertion of his authority in cases where the the patient offensively manifests a perverse disposition to dictate as to the mode of treatment or operations, thus virtually casting an imputation on the professional skill and superiority of the operator, which, whether it be done intentionally or thoughtlessly, is certainly to be regarded as an indication of bad taste on the part of the individual, and should be met with a candid dignified rebuke.

Let it be observed however that there is a distinction to be made between persons of the class we have mentioned and those who in good faith and with a proper deference to the professional character of our avocation, consult with the dentist and express their preference in cases where it is reasonably admissible so to do. But if the dental practitioner would sustain his claim to a *professional* standing he must promptly and effectually resent every aspersion on his skill and competency whether it be made covertly and intentionally, or carelessly and from a lack of appreciation.

It is always desirable to avoid, on the one hand, a tendency to enter freely into description and explanations of the details of any proposed operation or to expatiate on the *modus operandi* of therapeutic agents, etc., etc., since it must be apparent that such a course tends to detract from the importance and dignity of the profession however much it may appear to interest and entertain the auditor. On the other hand it is sometimes advisable and expedient to enlighten the patient, telling him why such and such a course of treatment is pursued, and to achieve what results; since it may be necessary, to insure a careful observance of instructions. But to enter further into details would perhaps tax the patience of my audience without resulting in the development of any ideas but such as have occurred to the minds of all present.

These ideas, gentlemen of the association, which I have but imperfectly embodied in this essay, which I shall not endeavor further to elaborate, may serve to suggest other and more important reflections pertaining to the subject under consideration. In these times of universal competition, he is blindly oblivious of his own interests who consents to ignore the existence of auxiliaries so invaluable as those which owe their origin to the cultivation of those qualities which recommend the man as a man, and a gentleman to his patients.

